# Photodynamic Therapy and Non-Melanoma Skin Cancer

**DOI:** 10.3390/cancers8100098

**Published:** 2016-10-22

**Authors:** Liezel L. Griffin, John T. Lear

**Affiliations:** Dermatology Centre, Salford Royal NHS Foundation Trust, Manchester Academic Health Science Centre, University of Manchester, Manchester M6 8HD, UK; liezel.griffin@doctors.org.uk

**Keywords:** photodynamic therapy, non-melanoma skin cancer, basal cell carcinoma, actinic keratosis, field cancerization, organ transplant recipients, Gorlin syndrome

## Abstract

Non-melanoma skin cancer (NMSC) is the most common malignancy among the Caucasian population. Photodynamic therapy (PDT) is gaining popularity for the treatment of basal cell carcinoma (BCC), Bowen’s disease (BD) and actinic keratosis (AK). A topical or systemic exogenous photosensitiser, results in selective uptake by malignant cells. Protoporphyrin IX (PpIX) is produced then activated by the introduction of a light source. Daylight-mediated MAL (methyl aminolaevulinate) PDT for AKs has the advantage of decreased pain and better patient tolerance. PDT is an effective treatment for superficial BCC, BD and both individual and field treatment of AKs. Excellent cosmesis can be achieved with high patient satisfaction. Variable results have been reported for nodular BCC, with improved outcomes following pretreatment and repeated PDT cycles. The more aggressive basisquamous, morphoeic infiltrating subtypes of BCC and invasive squamous cell carcinoma (SCC) are not suitable for PDT. Prevention of “field cancerization” in organ transplant recipients on long-term immunosuppression and patients with Gorlin syndrome (naevoid basal cell carcinoma syndrome) is a promising development. The optimisation of PDT techniques with improved photosensitiser delivery to target tissues, new generation photosensitisers and novel light sources may expand the future role of PDT in NMSC management.

## 1. Introduction

Non-melanoma skin cancer (NMSC) is the most common malignancy among the Caucasian population [1]. The incidence is estimated to rise by 3%–10% annually [2], presenting an increasing demand on healthcare resources. Surgery is the traditional mainstay of treatment, giving high cure rates with clear identification of tumour margins [3]. In recent decades, alternative chemical and physical destructive techniques have been introduced [4]. Photodynamic therapy (PDT) is gaining popularity for the treatment of certain types of basal cell carcinoma (BCC), Bowen’s disease (BD) and actinic keratoses (AK) [5].

PDT involves the application of an exogenous photosensitising agent, which is selectively taken up by malignant or premalignant cells [6]. The target cells convert the prodrug to protoporphyrin IX (PpIX), via the haem synthesis pathway [7]. Introduction of a light source causes activation of PpIX, formation of reactive oxygen species and cytotoxicity of malignant cells. Inflammation, secondary to PDT, induces proinflammatory cytokines, causing neutrophil migration to the treated tumour cells [8]. 

## 2. Topical PDT

The most commonly used topical photosensitisers are 5-aminolevulinic acid (ALA) and methyl aminolaevulinate (MAL) [6]. MAL is lipophilic and may offer benefits over ALA by greater penetration and specificity for target cells [9]. 

PpIX absorbs the greatest amount of light at the 410 nm wavelength, in the blue region. In practice, however, PDT light sources more commonly use 630 nm in the red region, giving better tissue penetration [5]. MAL is usually applied for 3 h before exposure to the light source and ALA is licensed for 14–18 h, although shorter periods are often observed [5,10].

First described by Slaughter in 1953, “field cancerization” refers to molecular changes from mutations of the p53 tumour suppressor gene. This results in subclinical malignant potential following conventional treatment for some epithelial tumours, including NMSC [11]. Topical PDT offers the advantage of field treatment to reduce the risk of further primary malignancies or recurrence. 

## 3. Adverse Effects

The main limitation of conventional PDT is burning and pain, which can be intolerable for some patients [12]. Treatment of facial AKs may result in greater pain than scalp lesions [13]. Localised skin reactions causing oedema and erythema also commonly occur, although scarring is rare [5]. 

## 4. Systemic PDT

Systemic PDT follows a similar method, using intravenous photosensitisers, followed by the application of a light source [14]. This allows deeper penetration of tumours, a key limitation of topical PDT. Porfimer-sodium (Photofrin^®^) was the first approved systemic photosensitiser and is indicated for lung and oesophageal malignancies [15]. The long half-life of Photofrin^®^ (21.5 days) results in generalised photosensitivity for several weeks, leading to poor patient tolerance and greater intensity of adverse skin reactions. 

Second generation agents, such as Temporfin (Foscan mTHPC) and silicon phthalocyanine (Pc4) have greater absorption at longer wavelengths, with shorter periods of generalised photosensitivity [15]. Verteporfin (Visudyne BPD-MA) has been investigated in an open-label, randomised study of 54 patients with nodular and superficial BCC and BD [14]. Following a single intravenous infusion and 180 J/cm^2^ of red light, 95% complete clearance rates were observed at 24 months. Less favourable outcomes were seen at lower light intensities (51% complete clearance with 60 J/cm^2^) [14]. Meta-tetra (hydroxyphenyl) chlorin (mTHPC) is a chlorophyll derivative, currently licensed for advanced head and neck squamous cell carcinoma (SCC) and its efficacy for NMSC remains to be determined [16]. 

## 5. Prophylactic PDT

Both topical and systemic PDT has shown benefit in murine studies for prophylaxis of NMSC [17,18,19]. Stender et al. investigated prevention of carcinogenesis using weekly topical ALA-PDT in hairless mice exposed to ultraviolet UV radiation. Despite delay in tumour development, the ALA treated group also experienced greater occurrence of large tumours (≥4 mm) [17]. This finding has not been corroborated in later studies. Liu et al. demonstrated that weekly topical or systemic PDT could increase tumour free survival on large skin surfaces of UV exposed hairless mice [18]. More recently, prophylactic treatment with either topical hexyl aminolevulinate (HAL 2%, 6% and 20%) or MAL (20%) were found to delay UV-induced SCC occurrence in hairless mice by 264 and 269 days respectively [19]. 

## 6. Daylight PDT

Daylight-mediated MAL-PDT allows field treatment of non-hyperkeratotic actinic keratoses [20]. This simplified method is effective as the red or blue wavelengths required to activate porphyrins are present in daylight [21]. Following pretreatment (e.g., abrasion or superficial curettage), the photosensitiser (MAL) is applied for 30 min [12]. Occlusion is not required [22]. Sunscreen without physical blocking filters is essential to protect exposed areas from ultra-violet damage [22]. 

The PpIX light dose must be equal to or greater than 8 J·cm^−2^ with an ambient temperature exceeding 10 °C [23]. Daylight PDT therefore remains suitable for use at higher latitudes, with seasonal limitation [23]. Patients are exposed to 2 h of daylight and must avoid sunlight for the remainder of the 24 h period [22].

Decreased pain and better tolerance compared to conventional PDT (cPDT) results from lower intensity continuous production of PpIX [24]. Similar or greater efficacy than cPDT has been reported in several international randomised studies [21,24,25,26]. One large Phase III multi-centre study reported 89.2% clearance for mild AKs at 12 weeks vs. 92.8% for MAL-cPDT [26]. A large European multi-centre Phase III, randomized, intra-individual study demonstrated non-inferiority of daylight PDT (70% compared to 74% clearance for cPDT of mild to moderate AKs at 12 weeks). Outcomes for daylight PDT were independent of weather conditions [24]. In a randomised multi-centre Nordic study, Wiegell et al. reported significantly greater efficacy for facial compared to scalp AKs treated with MAL daylight-PDT. Response rates were found to be independent of the overall mean effective daylight dose for all patients (9.4 J·cm^−2^) [27]. Smaller studies also support the use of daylight PDT. Clearance rates of 87% compared to 91% for cPDT were observed for grade I (mild) AKs in a prospective single-centre study [28]. Interestingly, a significantly increased clearance (up to 3%) was reported for each 5 °C rise in ambient temperature, thought to be due to increased production of PpIX within cells [28]. 

Little evidence exists regarding the use of ALA for daylight PDT. A small randomised prospective study comparing ALA nanoemulsion (BF-200 ALA) with MAL-PDT reported no significant results [29]. 

## 7. Basal Cell Carcinoma

PDT is an established treatment for superficial and nodular BCC, but is not indicated for the more aggressive basisquamous, morphoeic or infiltrating subtypes [30]. 

**Figure 1:**
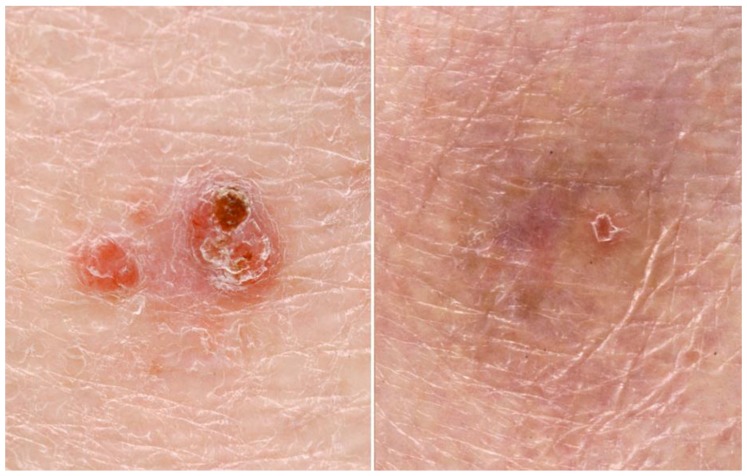
Superficial basal cell carcinoma before and after treatment with photodynamic therapy.

Szeimies et al. reported similar efficacy at 3 months for MAL-PDT and surgical excision in the management of superficial BCC in a large randomised multi-centre open study (92.2% clinical lesion response vs. 99.2% in the surgical group) [31]. Higher recurrence rates were observed following PDT, but the cosmetic outcome was superior [31]. A further randomised, multi-centre study compared MAL-PDT with cryotherapy for primary superficial BCC. There were comparable recurrence rates at 5 years, but a better cosmetic outcome was again observed for the MAL-PDT group [32]. In contrast, imiquimod has shown superiority and fluorouracil non-inferiority to MAL-PDT at 3 year follow-up of superficial BCC treatment in a large randomised controlled trial [33]. Improved clinical outcomes were found with repeated PDT cycles for primary superficial BCC in a recent systematic review (pooled complete tumour response increase from 75.6% to 79%) [34] (Figure 1).

Variable response rates for nodular BCC have been reported in several studies. MAL-PDT showed a significantly superior histological outcome to placebo at 6 months; with 73% to 78% complete response, compared to 27% to 33% for the placebo groups respectively in 2 multi-centre, double blind, randomised studies [35,36]. Rhodes et al. observed comparable responses for primary nodular BCC treated with MAL-PDT or surgical excision at 3 months (91% and 98% respectively), with greater recurrence rates but improved cosmesis in the PDT group [9]. Sustained complete lesion response rates at 5 years for surgical excision of nodular BCC, compared to MAL-PDT were reported in a later randomised study (96% vs. 76% respectively). PDT gave consistently better cosmetic outcomes [37]. In contrast, a multi-centre study by Fantini et al. reported complete response of only 33% for nodular BCC following two treatments of MAL-PDT, compared to 82% clearance of superficial BCC (mean follow up 23.5 months). Tumour thickness, ulceration and location were identified as a prognostic indicators, with higher cure rates for truncal compared to limb lesions [3]. A further randomised controlled study of 173 primary nodular BCCs found surgical excision to be significantly more effective compared to a single treatment of fractionated ALA-PDT, with a failure rate of 2.3% compared to 30.3% for PDT at 3 year follow up [38]. Soler et al. observed better outcomes using two ALA-PDT treatments for both nodular and superficial BCC, giving comparable clinical response rates to surgery (95.83% complete response cf. 95.65%). Recurrence rates were also similar (4.16% vs. 4.34%) [39]. Long-term recurrence may limit the use of PDT for nodular BCC, although it may be suitable for cases where surgical excision is not appropriate.

Greater frequency of recurrence is observed for more aggressive BCCs, which may be due to genetic mutations conferring resistance to apoptosis. Aggressive subtypes often occur on the face and PDT should therefore be used with caution for facial tumours [30]. Randomised studies with only short-term follow up had previously reported high efficacy for facial nodular BCCs treated with MAL-PDT [35]. 

The limited penetration of photosensitisers (1–2 mm) reduces the efficacy of PDT in thicker tumours. No association has been found between superficial BCC tumour thickness (up to 1 mm) and PDT failure [40]. Deep curettage prior to PDT may be beneficial for selected tumours, with cosmetic results maintained [41]. Dimethylsulphoxide (DMSO), which alters the intercellular lipid structure of the stratum corneum, has also been used as a pretreatment penetration enhancer [42]. Favourable 10-year response rates of 75% for primary small BCC have been achieved with curettage and DMSO pretreatment using ALA PDT for one or two sessions [43]. Intralesional ALA and light source application showed promising results in a small prospective study of 20 patients with nodular BCC, with no clinical recurrence observed (mean follow up 19.5 months) [44]. Pretreatment of nodular facial BCC with an ablative fractional laser is not currently recommended as an adjunctive therapy [45]. Preliminary studies have shown some benefit from a combination of PDT with Mohs micrographic surgery to reduce tumour size and improve cosmesis [46,47].

## 8. Actinic Keratosis

Actinic keratoses (AKs) are premalignant disorders of keratinocytes occurring on chronically sun-damaged skin. The risk of transformation to SCC in 12 months is between 0.025% and 16% [48]. Spontaneous regression may occur in up to 20% of cases [49]. Multiple AKs are often present and the risk of malignant transformation for individual lesions cannot be determined [50]. Treatment options include cryotherapy, topical immunomodulation, laser and PDT [51]. PDT is a highly effective treatment for AKs, recommended by international guidelines for both individual lesions and field treatment [5,52]. 

Tschen et al. reported clearance rates of 78% 12 months after a single ALA-PDT treatment in a Phase IV multicentre study, with few adverse effects [53]. Topical PDT has achieved favourable outcomes in comparison to cryotherapy in some studies [54,55]. After 12 weeks, novel ALA patch PDT demonstrated superiority to cryotherapy and placebo in a multi-centre Phase III trial [54]. Freeman et al. also observed significantly better lesion clearance rates with MAL-PDT compared to single cycle cryotherapy and placebo at 3 months in a further large prospective randomised study [55]. Despite better outcomes for MAL-PDT at 12 weeks in comparison to double freeze-thaw cryotherapy (86.9% vs. 76.2%), similar efficacy was reported at 24 weeks for face and scalp AKs in a large multi-centre randomised controlled study (89.1% lesion reduction with MAL-PDT, compared to 86.1% for cryotherapy) [50]. Further, Kaufmann et al. reported inferior efficacy of MAL-PDT vs. double cycle cryotherapy for AKs on the extremities in a large randomised multi-centre study (78% and 88% respectively, *p* = 0.002 per protocol population) [56]. However, superior cosmesis and patient satisfaction is an important advantage of PDT in the management of AKs [50,54,55]. 

An early randomised paired comparison of single ALA-PDT and topical 5-FU twice daily for 3 weeks found similar outcomes in mean lesion reduction (73% compared to 70%) after 6 months [57]. No significant difference was found in treatment response of facial AKs to topical 5% imiquimod compared to ALA-PDT in a randomised, single-blind, split-face study (n = 50) [58]. Sotiriou et al. observed comparable outcomes for ALA-PDT and 5% imiquimod for Grade 1 (mild) lesions on upper extremities, although ALA-PDT was significantly more effective for Grade 2 (moderate) AKs (57.89% response rate vs. 37.03% for imiquimod) [59]. However, imiquimod showed histological and clinical superiority over MAL-PDT for face and scalp AKs, in a further randomised study of 105 patients [60]. The same study found sequential MAL-PDT and imiquimod 5% significantly more effective than either therapy alone, indicating that combination treatment may be beneficial. An intra-individual comparison of ALA PDT with CO_2_ laser for the treatment of scalp AKs reported superior efficacy for PDT [61]. 

Improved uptake of photosensitiser and light penetrance can be achieved for hyperkeratotic AKs by physical or chemical keratolytic pretreatment [62]. Topical 10% salicylic acid and 40% urea have similar efficacy to curettage, although chemical pretreatment is associated with increased pain [62]. Ablative fractional laser resurfacing pretreatment may be beneficial for field treatment of moderate to severe AKs [49,63,64]. The recent introduction of ALA patch PDT reduces the need for prior debulking of hyperkeratotic AKs [65]. BF-200 ALA is a novel nanoemulsion formulation, which improves the stability of ALA while maintaining efficacy [66]. 

## 9. Bowen’s Disease

BD (SCC in situ) may evolve to invasive SCC in 3–5% of cases [67]. PDT has proven efficacy for treating BD and is recommended for both extensive involvement and poor healing sites [5]. Morton et al. compared MAL-PDT with cryotherapy and 5-FU in a placebo-controlled European multi-centre randomised study of 225 patients [68]. A significantly superior complete response rate was observed at 12 months for MAL-PDT compared to cryotherapy (80% vs. 67%, *p* = 0.047). Superior cosmesis was achieved with PDT [68]. ALA-PDT has also been found to be significantly more effective for BD than topical 5-FU at 12 months (82% vs. 48% complete clearance at 12 months, *p* = 0.006) [69]. A small prospective study of 23 biopsy proven extensive BD lesions (>3 cm) achieved 90% clearance with two MAL-PDT treatments, one week apart. Cosmetic outcome at 1 year was good or excellent, with recurrence in only three cases [70]. High total clearance rates (76.09% at 16.61 months) with two MAL-PDT treatments one week apart were reported in a retrospective, observational study of 51 BD lesions, with excellent cosmesis and only mild cutaneous adverse effects [71]. PDT appears to be a highly effective treatment for BD, however, long term follow up data is not yet available. Severe atypia in BD has been associated with a significantly poorer response to ALA-PDT [72]. Further investigation into the role of immunohistochemical factors in the response of BD to MAL-PDT is warranted. A small preliminary study has indicated that p53 and Ki67 expression could be markers of a positive response, although significance was not achieved [73]. 

## 10. Squamous Cell Carcinoma

PDT is not approved for invasive SCC (iSCC). Complete response rates of 73.2% at 3 months and 53.6% at 2 years have been reported [74]. The degree of cellular atypia contributes to the poor response. This was thought to be due to either reduced sensitivity to phototoxicty or decreased production of PpIX by undifferentiated keratinocytes [74]. Resistance occurs in a proportion of the exceptional iSCC cases treated with PDT, resulting in more aggressive disease. This effect may be due to chromosomal instability, which has been shown to cause overexpression of CCND1 and aberration of the MAPK/ERK signal pathway in immunodeficient mice [75]. 

## 11. Organ Transplant Recipients

Organ transplant recipients (OTR) have a greater risk of developing NMSC, in part due to the need for long-term immunosuppression [76]. Transplant procedures have also increased due to the ageing population and improved surgical techniques [77]. NMSC represents 95% of cutaneous malignancies in this patient group. The BCC:SCC ratio (4:1) seen in the immunocompetent population is reversed [78]. Higher mortality rates are observed, due to greater frequency and recurrence of lesions. Renal transplant patients have an 82% chance of developing SCC after 20 years [79]. Proactive, multi-disciplinary management of this patient group is challenging. 

PDT is an effective treatment for NMSC in OTRs, despite immunosuppressive therapy limiting proinflammatory cytokine recruitment [76]. A randomised, multi-centre study of 81 OTRs with 881 NMSCs (mainly AKs) compared MAL-PDT to standard treatment (either curettage, cryotherapy, surgery or laser). At 15 months, significantly fewer new AKs were found in the MAL-PDT treated area [80]. A further retrospective study found 77.4% clearance using two treatments of MAL-PDT, one week apart. No significant difference was found in recurrence rates of NMSC between OTRs and non-transplant recipients [81]. This was supported by a prospective study of superficial and nodular BCCs in 18 OTRs. Recurrence was observed in only one case following MAL-PDT with a mean follow up of 22.6 months [82]. However, despite similar results at 4 weeks, Dragieva et al. found much lower complete response rates at 48 weeks for AK and BD in OTRs (48%) compared to immunocompetent patients (72%) [83].

AK management in the OTR patient population is difficult. “Field cancerization” results in multiple, hyperkeratotic AKs, which require pretreatment and are more likely to recur [76,83]. PDT may be useful as a preventative measure in this patient group. An overall complete response rate of 56 out of 62 AKs following two MAL-PDT treatments one week apart was reported by Dragieva et al. in a randomised, placebo controlled, double blind study of 17 OTRs [84]. A randomised, single blinded, intra-individual trial of 25 renal transplant patients recently investigated repeated sessions of prophylactic PDT for prevention of field cancerization. After 3 years of follow up, there were significantly fewer AKs in PDT-treated compared to non-treated skin (8 vs. 43 respectively, *p* = 0.002) [85]. These findings were supported by Wulf et al. in a randomised pilot study of 27 renal OTRs, showing a significantly longer mean time to occurrence of new lesions in areas treated with MAL-PDT [86]. A small pilot study of cyclical ALA PDT reported benefit in the prevention of invasive and in situ SCC in organ transplant recipients (79.0% and 95% reduction in baseline SCC lesion count at 12 months and 24 months respectively). However, the study was limited by the small sample size (n = 12) and pretreatment of SCCs prior to commencing PDT [87]. Cyclical PDT may be beneficial for the prevention of AKs and SCC in this high-risk population, but further randomised studies with extended follow up are needed.

## 12. Gorlin Syndrome

Gorlin syndrome (naevoid basal cell carcinoma syndrome) is an autosomal dominant mutation of the PTCH-1 gene of the hedgehog signaling pathway, resulting in multiple early BCCs [88]. Surgical excision is the traditional gold standard of treatment, with the potential for significant scarring and disfigurement [89]. Adverse effects and variable response rates have limited alternative management options. Radiotherapy is contraindicated due to a paradoxical increase in BCCs related to the PTCH mutation [89]. Several small studies have supported the use of MAL-PDT for patients with Gorlin syndrome. Favourable cosmetic results have been achieved, with complete clearance in many cases [88,89,90,91,92,93]. 

Systemic PDT using Photofrin^®^ has shown benefit in a small case series. Madan et al. found 74.2% clearance of thicker BCCs following one treatment of Photofrin^®^ (n = 7). Interstitial optic diffuser fibres used in addition to a light source increased clearance to 87.6% in two cases [92]. Loncaster et al. studied systemic (Photofrin^®^) and topical (ALA or MAL) PDT in 33 Gorlin syndrome patients, using ultrasound to accurately assess tumour thickness and recurrence [89]. Complete clearance at 12 months was only 56.3%, which could reflect the greater sensitivity of ultrasound in detecting sub-clinical recurrence [89].

MAL-PDT is now approved in several countries for treating superficial and nodular BCC in this patient group [94]. It is also suitable for paediatric patients and the use of ropivacaine-lidocaine tumescent anaesthesia has been reported to increase tolerability [88]. A European consensus on MAL-PDT in Gorlin syndrome was published in 2014. This recommended MAL-PDT as a safe and effective alternative to surgery for all superficial BCCs and nodular BCCs <2 mm thickness [94]. 

## 13. Future Directions

New strategies for improving the efficacy and tolerability of PDT are under continuous development. Several classes of novel photosensitisers, for example fullerenes and phenothiazines, have been proposed [95]. The potential of low dose lipophilic Hexyl-5-aminolaevulinate (HAL) 0.2% to provide deeper penetration was investigated in comparison to MAL-PDT in a randomised pilot study. Comparable results were found for mild AKs, but HAL was less effective for moderate to severe lesions [96]. Better uptake and targeting of photosensitisers may be achieved in the future, using novel delivery systems such as nanoparticles, micelles or liposomes [95]. 

Pain is a key limiting factor to cPDT and the advent of daylight PDT is a promising development. The use of alternative light sources to decrease pain is also being investigated. Light emitting diodes (LED) deliver low irradiance and have shown good efficacy for treating BD, superficial BCC and AKs in preliminary studies [97,98]. Outcomes for pain control have been variable and the true benefit is yet to be determined. LED is lightweight and suitable for ambulatory PDT, which may provide greater convenience for patients [97]. New indications for PDT currently under exploration include cutaneous infections, inflammatory dermatoses, cutaneous T-cell lymphoma and extra-mammary Paget’s disease. Treatment of skin photoageing is also under investigation [99].

## 14. Conclusions

PDT has proven efficacy for certain types of NMSC (AK, BD, superficial BCC), with the benefit of excellent cosmetic results and the potential for field treatment. PDT should be used with caution for nodular BCC. Prophylactic PDT and field treatment for specialised patient groups, such as organ transplant recipients, are promising developments. The optimisation of techniques with daylight PDT, improved photosensitiser delivery to target tissues, new generation photosensitisers and novel light sources may expand the role of PDT in NMSC management in the future. 

## References

[1] Lomas A., Leonardi-Bee J., Bath-Hextall F. (2012). A systematic review of worldwide incidence of nonmelanoma skin cancer. Br. J. Dermatol..

[2] Roewert-Huber J., Lange-Asschenfeldt B., Stockfleth E., Kerl H. (2007). Epidemiology and aetiology of basal cell carcinoma. Br. J. Dermatol..

[3] Fantini F., Greco A., Del Giovane C., Cesinaro A.M., Venturini M., Zane C., Surrenti T., Peris K., Calzavara-Pinton P.G. (2011). Photodynamic therapy for basal cell carcinoma: Clinical and pathological determinants of response. J. Eur. Acad. Dermatol. Venereol..

[4] Griffin L.L., Ali F.R., Lear J.T. (2016). Non-melanoma skin cancer. Clin. Med..

[5] Morton C.A., Szeimies R.M., Sidoroff A., Braathen L.R. (2013). European guidelines for topical photodynamic therapy part 1: Treatment delivery and current indications actinic keratoses, Bowen's disease, basal cell carcinoma. J. Eur. Acad. Dermatol. Venereol..

[6] Szeimies R.M., Morton C.A., Sidoroff A., Braathen L.R. (2005). Photodynamic therapy for non-melanoma skin cancer. Acta Derm. Venereol..

[7] Kennedy J.C., Pottier R.H., Pross D.C. (1990). Photodynamic therapy with endogenous protoporphyrin. IX. basic principles and present clinical-experience. J. Photochem. Photobiol..

[8] Gollnick S.O., Evans S.S., Baumann H., Owczarczak B., Maier P., Vaughan L., Wang W.C., Unger E., Henderson B.W. (2003). Role of cytokines in photodynamic therapy-induced local and systemic inflammation. Br. J. Cancer.

[9] Rhodes L.E., de Rie M., Enstrom Y., Groves R., Morken T., Goulden V., Wong G.A.E., Grob J.J., Varma S., Wolf P. (2004). Photodynamic therapy using topical methyl aminolevulinate vs surgery for nodular basal cell carcinoma—Results of a multicenter randomized prospective trial. Archives Dermatol..

[10] Brown S.B., Brown E.A., Walker I. (2004). The present and future role of photodynamic therapy in cancer treatment. Lancet Oncol..

[11] Braakhuis B.J.M., Tabor M.P., Kummer J.A., Leemans C.R., Brakenhoff R.H. (2003). A genetic explanation of Slaughter’s concept of field cancerization: Evidence and clinical implications. Cancer Res..

[12] Braathen L.R. (2014). Photodynamic therapy: Increasing acceptance through reduction of adverse reactions. Br. J. Dermatol..

[13] Buinauskaite E., Zalinkevicius R., Buinauskiene J., Valiukeviciene S. (2013). Pain during topical photodynamic therapy of actinic keratoses with 5-aminolevulinic acid and red light source: Randomized controlled trial. Photodermatol. Photoimmunol. Photomed..

[14] Lui H., Hobbs L., Tope W.D., Lee P.K., Elmets C., Provost N., Chan A., Neyndorff H., Su X.Y., Jain H. (2004). Photodynamic therapy of multiple nonmelanoma skin cancers with verteporfin and red light-emitting diodes—Two-year results evaluating tumor response and cosmetic outcomes. Arch. Dermatol..

[15] Dabrowski J.M., Arnaut L.G. (2015). Photodynamic therapy (PDT) of cancer: from local to systemic treatment. Photochem. Photobiol. Sci..

[16] Horlings R.K., Terra J.B., Witjes M.J.H. (2015). mTHPC mediated, systemic photodynamic therapy (PDT) for nonmelanoma skin cancers: Case and literature review. Lasers Surg. Med..

[17] Stender I.M., BechThomsen N., Poulsen T., Wulf H.C. (1997). Photodynamic therapy with topical delta-aminolevulinic acid delays UV photocarcinogenesis in hairless mice. Photochem. Photobiol..

[18] Liu Y.N., Viau G., Bissonnette R. (2004). Multiple large-surface photodynamic therapy sessions with topical or systemic aminolevulinic acid and blue light in UV-exposed hairless mice. J. Cutan. Med. Surg..

[19] Togsverd-Bo K., Lerche C.M., Poulsen T., Wulf H.C., Haedersdal M. (2010). Photodynamic therapy with topical methyl- and hexylaminolevulinate for prophylaxis and treatment of UV-induced SCC in hairless mice. Exp. Dermatol..

[20] Wiegell S.R., Wulf H.C., Szeimies R.M., Basset-Seguin N., Bissonnette R., Gerritsen M.J.P., Gilaberte Y., Calzavara-Pinton P., Morton C.A., Sidoroff A. (2012). Daylight photodynamic therapy for actinic keratosis: An international consensus. J. Eur. Acad. Dermatol. Venereol..

[21] Wiegell S.R., Haedersdal M., Philipsen P.A., Eriksen P., Enk C.D., Wulf H.C. (2008). Continuous activation of PpIX by daylight is as effective as and less painful than conventional photodynamic therapy for actinic keratoses; a randomized, controlled, single-blinded study. Br. J. Dermatol..

[22] Philipp-Dormston W.G., Sanclemente G., Torezan L., Clementoni M.T., Le Pillouer-Prost A., Cartier H., Szeimies R.M., Bjerring P. (2016). Daylight photodynamic therapy with MAL cream for large-scale photodamaged skin based on the concept of "actinic field damage": Recommendations of an international expert group. J. Eur. Acad. Dermatol. Venereol..

[23] Wiegell S.R., Fabricius S., Heydenreich J., Enk C.D., Rosso S., Baeumler W., Baldursson B.T., Wulf H.C. (2013). Weather conditions and daylight-mediated photodynamic therapy: protoporphyrin IX-weighted daylight doses measured in six geographical locations. Br. J. Dermatol..

[24] Lacour J.P., Ulrich C., Gilaberte Y., Von Felbert V., Basset-Seguin N., Dreno B., Girard C., Redondo P., Serra-Guillen C., Synnerstad I. (2015). Daylight photodynamic therapy with methyl aminolevulinate cream is effective and nearly painless in treating actinic keratoses: A randomised, investigator-blinded, controlled, phase III study throughout Europe. J. Eur. Acad. Dermatol. Venereol..

[25] Cantisani C., Paolino G., Cantoresi F., Calvieri S. (2015). Daylight photodynamic therapy for the treatment of actinic keratosis. J. Am. Acad. Dermatol..

[26] Rubel D.M., Spelman L., Murrell D.F., See J.A., Hewitt D., Foley P., Bosc C., Kerob D., Kerrouche N., Wulf H.C. (2014). Daylight photodynamic therapy with methyl aminolevulinate cream as a convenient, similarly effective, nearly painless alternative to conventional photodynamic therapy in actinic keratosis treatment: A randomized controlled trial. Br. J. Dermatol..

[27] Wiegell S.R., Fabricius S., Stender I.M., Berne B., Kroon S., Andersen B.L., Mork C., Sandberg C., Jemec G.B.E., Mogensen M. (2011). A randomized, multicentre study of directed daylight exposure times of 11/2 vs. 21/2 h in daylight-mediated photodynamic therapy with methyl aminolaevulinate in patients with multiple thin actinic keratoses of the face and scalp. Br. J. Dermatol..

[28] Fargnoli M.C., Piccioni A., Neri L., Tambone S., Pellegrini C., Peris K. (2015). Conventional vs. daylight methyl aminolevulinate photodynamic therapy for actinic keratosis of the face and scalp: An intra-patient, prospective, comparison study in Italy. J. Eur. Acad. Dermatol. Venereol..

[29] Neittaanmaki-Perttu N., Karppinen T.T., Gronroos M., Tani T.T., Snellman E. (2014). Daylight photodynamic therapy for actinic keratoses: A randomized double-blinded nonsponsored prospective study comparing 5-aminolaevulinic acid nanoemulsion (BF-200) with methyl-5-aminolaevulinate. Br. J. Dermatol..

[30] Fiechter S., Skaria A., Nievergelt H., Anex R., Borradori L., Parmentier L. (2012). Facial basal cell carcinomas recurring after photodynamic therapy: A retrospective analysis of histological subtypes. Dermatology.

[31] Szeimies R.M., Ibbotson S., Murrell D.F., Rubel D., Frambach Y., de Berker D., Dummer R., Kerrouche N., Villemagne H. (2008). A clinical study comparing methyl aminolevulinate photodynamic therapy and surgery in small superficial basal cell carcinoma (8–20 mm), with a 12-month follow-up. J. Eur. Acad. Dermatol. Venereol..

[32] Basset-Seguin N., Ibbotson S.H., Emtestam L., Tarstedt M., Morton C., Maroti M., Calzavara-Pinton P., Varma S., Roelandts R., Wolf P. (2008). Topical methyl aminolaevulinate photodynamic therapy versus cryotherapy for superficial basal cell carcinoma: A 5 year randomized trial. Euro. J. Dermatol..

[33] Arits A.H.M.M., Mosterd K., Essers B.A.B., Spoorenberg E., Sommer A., de Rooij M.J.M., van Pelt H.P., Quaedvlieg P.J., Krekels G.A., van Neer P.A. (2013). Photodynamic therapy versus topical imiquimod versus topical fluorouracil for treatment of superficial basal-cell carcinoma: a single blind, non-inferiority, randomised controlled trial. Lancet Oncol..

[34] Roozeboom M.H., Arits A.H.H.M., Nelemans P.J., Kelleners-Smeets N.W.J. (2012). Overall treatment success after treatment of primary superficial basal cell carcinoma: A systematic review and meta-analysis of randomized and nonrandomized trials. Br. J. Dermatol..

[35] Foley P., Freeman M., Menter A., Siller G., El-Azhary R.A., Gebauer K., Lowe N.J., Jarratt N.T., Murrell D.F., Rich P. (2009). Photodynamic therapy with methyl aminolevulinate for primary nodular basal cell carcinoma: Results of two randomized studies. Inter. J. Dermatol..

[36] Tope W.D., Menter A., El-Azhary R.A., Lowe N.J. (2004). Comparison of topical methylaminolevulinate photodynamic therapy versus placebo photodynamic therapy in nodular basal cell carcinoma. J. Am. Acad. Dermatol..

[37] Rhodes L.E., de Rie M.A., Leifsdottir R., Yu R.C., Bachmann I., Goulden V., Wong G.A., Richard M.A., Anstey A., Wolf P. (2007). Five-year follow-up of a randomized, prospective trial of topical methyl aminolevulinate photodynamic therapy vs. surgery for nodular basal cell carcinoma. Arch. Dermatol..

[38] Mosterd K., Thissen M., Nelemans P., Kelleners-Smeets N.W.J., Janssen R., Broekhof K., Neumann H.A., Steijlen P.M., Kuijpers D.I. (2008). Fractionated 5-aminolaevulinic acid-photodynamic therapy vs. surgical excision in the treatment of nodular basal cell carcinoma: Results of a randomized controlled trial. Br. J. Dermatol..

[39] Cosgarea R., Susan M., Crisan M., Senila S. (2013). Photodynamic therapy using topical 5-aminolaevulinic acid vs. surgery for basal cell carcinoma. J. Eur. Acad. Dermatol. Venereol..

[40] Roozeboom M.H., van Kleef L., Arits A.H.M.M., Mosterd K., Winnepenninckx V.J.L., van Marion A.M.W., Nelemans P.J., Kelleners-Smeets N.W. (2015). Tumor thickness and adnexal extension of superficial basal cell carcinoma (sBCC) as determinants of treatment failure for methylaminolevulinate (MAL)-photodynamic therapy (PDT), imiquimod, and 5-fluorouracil (FU). J. Am. Acad. Dermatol..

[41] Christensen E., Mork C., Foss O.A. (2011). Pre-treatment deep curettage can significantly reduce tumour thickness in thick Basal cell carcinoma while maintaining a favourable cosmetic outcome when used in combination with topical photodynamic therapy. J. Skin Cancer.

[42] Gerritsen M.J.P., Smits T., Kleinpenning M.M., van de Kerkhof P.C.M., van Erp P.E.J. (2009). Pretreatment to Enhance Protoporphyrin IX Accumulation in Photodynamic Therapy. Dermatology.

[43] Christensen E., Mork C., Skogvoll E. (2012). High and sustained efficacy after two sessions of topical 5-aminolaevulinic acid photodynamic therapy for basal cell carcinoma: a prospective, clinical and histological 10-year follow-up study. Br. J. Dermatol..

[44] Angel Rodriguez-Prieto M., Gonzalez-Sixto B., Perez-Bustillo A., Alonso-Alonso T., Ortega-Valin L., Martinez-Valderrabano V., González-Morán A., García Doval I. (2012). Photodynamic therapy with intralesional photosensitizer and laser beam application: An alternative treatment for nodular basal cell carcinoma. J. Am. Acad. Dermatol..

[45] Haak C.S., Togsverd-Bo K., Thaysen-Petersen D., Wulf H.C., Paasch U., Anderson R.R., Haedersdal M. (2015). Fractional laser-mediated photodynamic therapy of high-risk basal cell carcinomas—A randomized clinical trial. Br. J. Dermatol..

[46] Kuijpers D.I.M., Smeets N.W.J., Krekels G.A.M., Thissen M. (2004). Photodynamic therapy as adjuvant treatment of extensive basal cell carcinoma treated with Mohs micrographic surgery. Dermatol. Surg..

[47] Al-Niaimi F., Sheth N., Kurwa H.A., Mallipeddi R. (2015). Photodynamic therapy followed by mohs micrographic surgery compared to mohs micrographic surgery alone for the treatment of basal cell carcinoma: Results of a pilot single-blinded randomised controlled trial. J. Cutan. Aesthet. Surg..

[48] Glogau R.G. (2000). The risk of progression to invasive disease. J. Am. Acad. Dermatol..

[49] Ko D.Y., Jeon S.Y., Kim K.H., Song K.H. (2014). Fractional erbium: YAG laser-assisted photodynamic therapy for facial actinic keratoses: A randomized, comparative, prospective study. J. Eur. Acad. Dermatol. Venereol..

[50] Morton C., Campbell S., Gupta G., Keohane S., Lear J., Zaki I., Walton S., Kerrouche N., Thomas G., Soto P. (2006). Intraindividual, right-left comparison of topical methyl aminolaevulinate-photodynamic therapy and cryotherapy in subjects with actinic keratoses: A multicentre, randomized controlled study. Br. J. Dermatol..

[51] Lucena S.R., Salazar N., Gracia-Cazana T., Zamarron A., Gonzalez S., Juarranz A., Gilaberte Y. (2015). Combined Treatments with Photodynamic Therapy for Non-Melanoma Skin Cancer. Int. J. Mol. Sci..

[52] Braathen L.R., Szeimies R.-M., Basset-Seguin N., Bissonnette R., Foley P., Pariser D., Roelandts R., Wennberg A.M., Morton C.A. (2007). Guidelines on the use of photodynamic therapy for nonmelanoma skin cancer: An international consensus. J. Am. Acad. Dermatol..

[53] Tschen E.H., Wong D.S., Pariser D.M., Dunlap F.E., Houihan A., Ferdon M.B., the Phase IV ALA-PDT Actinic Keratosis Study Group (2006). Photodynamic therapy using aminotaevulinic acid for patients with nonhyperkeratotic actinic keratoses of the face and scalp: Phase IV multicentre clinical trial with 12-month follow up. Br. J. Dermatol..

[54] Hauschild A., Stockfleth E., Popp G., Borrosch F., Bruening H., Dominicus R., Mensing H., Reinhold U., Reich K., Moor A.C. (2009). Optimization of photodynamic therapy with a novel self-adhesive 5-aminolaevulinic acid patch: Results of two randomized controlled phase III studies. Br. J. Dermatol..

[55] Freeman M., Vinciullo C., Francis D., Spelman L., Nguyen R., Fergin P., Thai K.E., Murrell D., Weightman W., Anderson C. (2003). A comparison of photodynamic therapy using topical methyl aminolevulinate (Metvix) with single cycle cryotherapy in patients with actinic keratosis: A prospective, randomized study. J. Dermatol. Treat..

[56] Kaufmann R., Spelman L., Weightman W., Reifenberger J., Szeimies R.M., Verhaeghe E., Kerrouche N., Sorba V., Villemagne H., Rhodes L.E. (2008). Multicentre intraindividual randomized trial of topical methyl aminolaevulinate-photodynamic therapy vs. cryotherapy for multiple actinic keratoses on the extremities. Br. J. Dermatol..

[57] Kurwa H.A., Yong-Gee S.A., Seed P.T., Markey A.C., Barlow R.J. (1999). A randomized paired comparison of photodynamic therapy and topical 5-fluorouracil in the treatment of actinic keratoses. J. Am. Acad. Dermatol..

[58] Hadley J., Tristani-Firouzi P., Hull C., Florell S., Cotter M., Hadley M. (2012). Results of an investigator-initiated single-blind split-face comparison of photodynamic therapy and 5% imiquimod cream for the treatment of actinic keratoses. Dermatol. Surg..

[59] Sotiriou E., Apalla Z., Maliamani F., Zaparas N., Panagiotidou D., Ioannides D. (2009). Intraindividual, right-left comparison of topical 5-aminolevulinic acid photodynamic therapy vs. 5% imiquimod cream for actinic keratoses on the upper extremities. J. Eur. Acad. Dermatol. Venereol..

[60] Serra-Guillen C., Nagore E., Hueso L., Traves V., Messeguer F., Sanmartin O., Llombart B., Requena C., Botella-Estrada R., Guillén C. (2012). A randomized pilot comparative study of topical methyl aminolevulinate photodynamic therapy versus imiquimod 5% versus sequential application of both therapies in immunocompetent patients with actinic keratosis: Clinical and histologic outcomes. J. Am. Acad. Dermatol..

[61] Scola N., Terras S., Georgas D., Othlinghaus N., Matip R., Pantelaki I., Möllenhoff K., Stücker M., Altmeyer P., Kreuter A. (2012). A randomized, half-side comparative study of aminolaevulinate photodynamic therapy vs. CO_2_ laser ablation in immunocompetent patients with multiple actinic keratoses. Br. J. Dermatol..

[62] Gholam P., Fink C., Bosselmann I., Enk A.H. (2016). Retrospective analysis evaluating the effect of a keratolytic and physical pretreatment with salicylic acid, urea and curettage on the efficacy and safety of photodynamic therapy of actinic keratoses with methylaminolaevulinate. J. Eur. Acad. Dermatol. Venereol..

[63] Togsverd-Bo K., Haak C.S., Thaysen-Petersen D., Wulf H.C., Anderson R.R., Haedesdal M. (2012). Intensified photodynamic therapy of actinic keratoses with fractional CO_2_ laser: A randomized clinical trial. Br. J. Dermatol..

[64] Song H.S., Jung S.-E., Jang Y.H., Kang H.Y., Lee E.-S., Kim Y.C. (2015). Fractional carbon dioxide laser-assisted photodynamic therapy for patients with actinic keratosis. Photodermatol. Photoimmunol. Photomed..

[65] Szeimies R.M., Hauschild A., Ortland C., Moor A.C.E., Stocker M., Surber C. (2015). Photodynamic therapy simplified: nonprepared, moderate-grade actinic keratosis lesions respond equally well to 5-aminolaevulinic acid patch photodynamic therapy as do mild lesions. Br. J. Dermatol..

[66] Szeimies R.M., Radny P., Sebastian M., Borrosch F., Dirschka T., Krahn-Senftleben G., Reich K., Pabst G., Voss D., Foguet M. (2010). Photodynamic therapy with BF-200 ALA for the treatment of actinic keratosis: results of a prospective, randomized, double-blind, placebo-controlled phase III study. Br. J. Dermatol..

[67] Gracia-Cazana T., Teresa Lopez M., Oncins R., Gilaberte Y. (2015). Successful treatment of sequential therapy in digital Bowen’s disease with methyl aminolevulinate photodynamic therapy and topical diclofenac 3% in hyaluronan 2.5% gel. Dermatol. Ther..

[68] Morton C., Horn M., Leman J., Tack B., Bedane C., Tjioe M., Ibbotson S., Khemis A., Wolf P. (2006). Comparison of topical methyl aminolevulinate photodynamic therapy with cryotherapy or fluorouracil for treatment of squamous cell carcinoma in situ—Results of a multicenter randomized trial. Arch. Dermatol..

[69] Salim A., Leman J.A., McColl J.H., Chapman R., Morton C.A. (2003). Randomized comparison of photodynamic therapy with topical 5-fluorouracil in Bowen's disease. Br. J. Dermatol..

[70] Lopez N., Meyer-Gonzalez T., Herrera-Acosta E., Bosch R., Castillo R., Herrera E. (2012). Photodynamic therapy in the treatment of extensive Bowen's disease. J. Dermatol. Treat..

[71] Truchuelo M., Fernandez-Guarino M., Fleta B., Alcantara J., Jaen P. (2012). Effectiveness of photodynamic therapy in Bowen’s disease: An observational and descriptive study in 51 lesions. J. Eur. Acad. Dermatol. Venereol..

[72] Westers-Attema A., Lohman B.G.P.M., van den Heijkant F., Nelemans P.J., Winnepenninckx V.J., Kelleners-Smeets N.W.J., Mosterd K. (2015). Photodynamic therapy in Bowen’s disease: Influence of histological features and clinical characteristics on its success. Dermatology.

[73] Gracia-Cazana T., Vera-Alvarez J., Juarranz A., Pastushenko I., Salazar N., Gonzalez S., Gilaberte Y. (2015). Clinicopathological features of Bowen’s disease resistance to methyl aminolevulinate photodynamic therapy. J. Investig. Dermatol..

[74] Calzavara-Pinton P.G., Venturini M., Sala R., Capezzera R., Parrinello G., Specchia C., Zane C. (2008). Methylaminolaevulinate-based photodynamic therapy of Bowen’s disease and squamous cell carcinoma. Br. J. Dermatol..

[75] Gilaberte Y., Milla L., Salazar N., Vera-Alvarez J., Kourani O., Damian A., Rivarola V., Roca M.J., Espada J., González S. (2014). Cellular Intrinsic factors involved in the resistance of squamous cell carcinoma to photodynamic therapy. J. Investig. Dermatol..

[76] Basset-Seguin N., Conzett K.B., Gerritsen M.J.P., Gonzalez H., Haedersdal M., Hofbauer G.F.L., Aguado L., Kerob D., Lear J.T., Piaserico S. (2013). Photodynamic therapy for actinic keratosis in organ transplant patients. J. Eur. Acad. Dermatol. Venereol..

[77] Wlodek C., Ali F.R., Lear J.T. (2013). Use of photodynamic therapy for treatment of actinic keratoses in organ transplant recipients. BioMed. Res. Int..

[78] Ulrich C., Kanitakis J., Stockfleth E., Euvrard S. (2008). Skin cancer in organ transplant recipients - Where do we stand today ?. Am. J. Transplant..

[79] Ramsay H.M., Fryer A.A., Hawley C.M., Smith A.G., Nicol D.L., Harden P.N. (2002). Non-melanoma skin cancer risk in the Queensland renal transplant population. Br. J. Dermatol..

[80] Wennberg A.M., Keohane S., T Lear J., Jemec G., Mork C., Christensen E., Kapp A., Solvsten H., Talm T., Berne B. (2006). Results from a 15-month update of a multicentre study of methyl aminolaevulinate photodynamic therapy in immunocompromised organ transplant recipients with nonmelanoma skin cancer. Br. J. Dermatol..

[81] Collier N.J., Ali F.R., Lear J.T. (2015). Efficacy of photodynamic therapy for treatment of basal cell carcinoma in organ transplant recipients. Lasers Med. Sci..

[82] Guleng G.E., Helsing P. (2012). Photodynamic therapy for basal cell carcinomas in organ-transplant recipients. Clin. Exp. Dermatol..

[83] Dragieva G., Hafner J., Dummer R., Schmid-Grendelmeier P., Roos M., Prinz B.M., Burg G., Binswanger U., Kempf W. (2004). Topical photodynamic therapy in the treatment of actinic keratoses and Bowen's disease in transplant recipients. Transplantation.

[84] Dragieva G., Prinz B.M., Hafner J., Dummer R., Burg G., Binswanger U., Kempf W. (2004). A randomized controlled clinical trial of topical photodynamic therapy with methyl aminolaevulinate in the treatment of actinic keratoses in transplant recipients. Br. J. Dermatol..

[85] Togsverd-Bo K., Omland S.H., Wulf H.C., Sorensen S.S., Haedersdal M. (2015). Primary Prevention of Skin Dysplasia in Renal Transplant Recipients With Photodynamic Therapy: A Randomized Controlled Trial. Am. J. Transplant..

[86] Wulf H.C., Pavel S., Stender I., Bakker-Wensveen C.A. (2006). Topical photodynamic therapy for prevention of new skin lesions in renal transplant recipients. Acta Derm. Venereol..

[87] Willey A., Mehta S., Lee P.K. (2010). Reduction in the incidence of squamous cell carcinoma in solid organ transplant recipients treated with cyclic photodynamic therapy. Dermatol. Surg..

[88] Girard C., Debu A., Bessis D., Blatiere V., Dereure O., Guillot B. (2013). Treatment of Gorlin syndrome (nevoid basal cell carcinoma syndrome) with methylaminolevulinate photodynamic therapy in seven patients, including two children: Interest of tumescent anesthesia for pain control in children. J. Eur. Acad. Dermatol. Venereol..

[89] Loncaster J., Swindell R., Slevin F., Sheridan L., Allan D., Allan E. (2009). Efficacy of Photodynamic Therapy as a Treatment for Gorlin Syndrome-related Basal Cell Carcinomas. Clin. Oncol..

[90] Fai D. (2006). MAL-PDT for the treatment of multiple basal cell carcinomas in a patient with Gorlin-Goltz syndrome. J. Invest. Dermatol..

[91] Neves D.R., Ramos D.G., Magalhaes G.M., Rodrigues R.d.C., Alves de Souza J.B. (2010). Photodynamic therapy for treatment of multiple lesions on the scalp in nevoid basal cell carcinoma syndrome—Case report. An. Bras. Dermatol..

[92] Madan V., Loncaster J., Allan D., Lear J., Sheridan L., Leach C., Allan E. (2005). Systemic photodynamic therapy with Photofrin for naevoid basal cell carcinoma syndrome—A pilot study. Photodiagnosis Photodyn. Ther..

[93] Mougel F., Debarbieux S., Ronger-Savle S., Dalle S., Thomas L. (2009). Methylaminolaevulinate Photodynamic therapy in patients with multiple basal cell carcinomas in the setting of gorlin-goltz syndrome or after radiotherapy. Dermatology.

[94] Basset-Seguin N., Bissonnette R., Girard C., Haedersdal M., Lear J.T., Paul C., Piaserico S. (2014). Consensus recommendations for the treatment of basal cell carcinomas in Gorlin syndrome with topical methylaminolaevulinate-photodynamic therapy. J. Eur. Acad. Dermatol. Venereol..

[95] Alexiades-Armenakas M. (2011). The future of photodynamic therapy. Photodyn. Ther. Dermatol..

[96] Neittaanmaki-Perttu N., Gronroos M., Karppinen T.T., Tani T.T., Snellman E. (2016). Hexyl-5-aminolaevulinate 0.2% vs. methyl-5-aminolaevulinate 16% daylight photodynamic therapy for treatment of actinic keratoses: Results of a randomized double-blinded pilot trial. Br. J. Dermatol..

[97] Attili S.K., Lesar A., McNeill A., Camacho-Lopez M., Moseley H., Ibbotson S., Samuel I.D., Ferguson J. (2009). An open pilot study of ambulatory photodynamic therapy using a wearable low-irradiance organic light-emitting diode light source in the treatment of nonmelanoma skin cancer. Br. J. Dermatol..

[98] Babilas P., Travnik R., Werner A., Landthaler M., Szeimies R.M. (2008). Split-face study using two different light sources for topical PDT of actinic keratoses: Non-inferiority of the LED-system. J. Dtsch. Dermatol. Ges..

[99] Morton C.A., Szeimies R.M., Sidoroff A., Braathen L.R. (2013). European guidelines for topical photodynamic therapy part 2: Emerging indications—Field cancerization, photorejuvenation and inflammatory/infective dermatoses. J. Eur. Acad. Dermatol. Venereol..

